# Comparative Analysis of Displacements and Stress Produced by Square and Rectangular Bracket Slot With Incremental Torque: A Finite Element Analysis Study

**DOI:** 10.7759/cureus.71528

**Published:** 2024-10-15

**Authors:** Sarvajith B Harsha, Khadeer Riyaz, Laxmikanth S Manjappa, Ashita Talwar, Divya Ravuru, Nandan K Narasimha Mogaveera, Seema Gupta

**Affiliations:** 1 Department of Orthodontics and Dentofacial Orthopedics, The Oxford Dental College, Bengaluru, IND; 2 Department of Orthodontics and Dentofacial Orthopedics, Kothiwal Dental College and Research Centre, Moradabad, IND

**Keywords:** bracket, conventional, cortical bone, finite element analysis, passive, stress

## Abstract

Introduction: This study aimed to perform a comparative analysis of stresses and displacements with incremental torque on the maxillary incisors and surrounding cortical bone using conventional metal brackets with rectangular slots and passive self-ligating brackets with square slots using finite element analysis (FEA).

Materials and methods: An in vitro FEA study was conducted, in which a three-dimensional (3D) model of the maxilla was built using ANSYS software version 18 (ANSYS Inc., Canonsburg, PA). The conventional McLaughlin, Bennet, and Trevisi (MBT) bracket (3M Unitek, Monrovia, CA) of 0.022 × 0.028-inch slot with a 0.019 × 0.025-inch rectangular stainless steel (SS) archwire (model 1) was compared with the Pitts 21™ self-ligating bracket system (OC Orthodontics, McMinnville, OR) of 0.021 x 0.21-inch slot with a 0.020 × 0.020-inch square SS archwire (model 2) at incremental torques of 5°, 10°, 15°, and 20°. The von Mises stress was evaluated at the maxillary incisors and surrounding cortical bone. The torque moment, tipping angle, and displacement of the maxillary incisors were also measured and compared between the bracket systems.

Results: The highest torque expression of 13.8 N-mm was observed in model 2, compared to a torque expression of 10.68 N-mm in model 1. The torque expression increased in both models from 0°to 20^°^. There was a play of 6.2°at 20°torque in model 2, whereas it was 9.32°​​​​​​​play in model 1. The torque expressions were better for the lateral incisors than for the central incisors. Increased incremental torque was associated with increased proclination of the incisors, and this movement was more pronounced for the central incisor and model 1. Furthermore, it was revealed that the stresses on the cortical bone and teeth were higher in model 2 than in model 1.

Conclusion: It was concluded that the Pitts 21™ passive self-ligating system produced better torque expression and less play with square SS archwire compared to conventional brackets with rectangular SS archwire.

## Introduction

Tooth inclination in the labiolingual direction is essential to achieve optimal occlusal interactions and maintain long-term treatment stability. The improper labiolingual inclination of the anterior teeth can lead to space deficiencies in the dental arch, hampering the establishment of a firm class I relationship, anterior guidance, and aesthetically pleasing smile characteristics. Likewise, the inadequate inclination of the posterior segment may disrupt cusp-to-fossa relationships between the maxillary and mandibular teeth [1]. Torque, which controls the axial tooth inclination, is crucial for aligning teeth in optimal positions. It is particularly important in the maxillary incisors, where it contributes to the appropriate interincisal angle, incisor contact, and sagittal alignment of the dentition, which are all essential for achieving ideal occlusion [2].

However, torque control involves complex biomechanical processes. Various factors, such as the wire's cross-sectional properties, edge bevel, bracket slot engagement angle, and interaction between the wire and tooth morphology, influence the extent to which the torque is expressed. This movement results from the interaction of localized pressure and tension generated in the alveolar bone owing to torsion in the archwire [3]. However, torque is often poorly understood, and improper application can lead to complications during orthodontic treatment.

The effective expression of torque relies on a precise fit between the archwire and the bracket slot. Careful diagnosis, treatment planning, and biomechanical execution are required for successful orthodontic outcomes. Particularly in straight-wire appliances, the accurate expression of bracket prescriptions and the mechanical properties of the archwire play a pivotal role. Clinicians often encounter challenges in achieving full torque expression owing to undersized wires or poor archwire-slot coupling [4]. Moreover, the torque expression is influenced by numerous factors, including bracket design, wire torsion, stiffness, and edge bevel [5]. Thus, achieving the desired tooth movement necessitates tight coupling between the archwire and bracket slot, which is crucial for anterior and posterior torque expression. This study aimed to evaluate and compare the fit and torque expression of stainless steel (SS) wire in square and rectangular orthodontic brackets using finite element analysis (FEA) to enhance the precision of orthodontic treatment.

## Materials and methods

Study design and setting

This in vitro simulation study was conducted at the Department of Orthodontics, Oxford Dental College, Bommanahalli, Bengaluru, from October 2022 to December 2023. This study aimed to analyze and compare the precise fit and torque expression of two different orthodontic bracket systems: the Pitts 21™ self-ligating bracket system (OC Orthodontics, McMinnville, OR) with a slot size of 0.021 × 0.021-inch and the conventional McLaughlin, Bennet, and Trevisi (MBT) rectangular bracket with a slot size of 0.022 × 0.028-inch (3M Unitek, Monrovia, CA). As this study was based on FEA using three-dimensional (3D) models on cone beam computed tomography (CBCT) scan of a patient that was already in the database, ethical approval was not required.

FEA model development

FEA was conducted using ANSYS software version 18 (ANSYS Inc., Canonsburg, PA). The finite element study examined the mechanical interactions between the bracket and archwire combinations. For the conventional MBT brackets, a 0.019 × 0.025-inch rectangular SS archwire was selected (model 1), and for the Pitts 21™ self-ligating bracket system, a 0.020 × 0.020-inch square SS archwire was used (model 2). These archwire sizes were specifically chosen to reflect the clinically relevant combinations. Torque angles of 5°, 10°, 15°, and 20° were applied to simulate real-world torque expressions. These torque angles represent the degree of rotation of the archwire within the bracket slot, mimicking the forces exerted during orthodontic treatment to control the tooth movement. Finite element models were designed to simulate mechanical interactions, such as stress distribution, friction, and deformation, under these different torque angles.

The study design followed several sequential steps to achieve accurate FEA simulation. First, 3D models of the maxillary right central and lateral incisors were developed, incorporating the details of the periodontal ligament (PDL) and alveolar bone. These models were based on the standard anatomical features of human dentition. Next, 3D representations of brackets and archwires were designed using computer-aided design/computer-aided manufacturing (CAD/CAM) data provided by the manufacturer. These models included both the bracket system and the respective torque prescriptions at 5°, 10°, 15°, and 20°.

Finally, the torque expression capabilities of each archwire-bracket combination were analyzed under different torque angles. The developed 3D models were subjected to FEA simulations to determine the stress distribution and magnitude across the archwire-bracket combinations.

3D model of incisors

A 3D model of the maxillary right central and lateral incisors, including the surrounding periodontal ligament (PDL) and alveolar bone, was developed. The PDL was modeled with a uniform thickness of 0.2 mm, as well as the alveolar bone with a uniform thickness of 0.5 mm. These geometries are based on a commercial 3D dataset, ensuring an anatomically accurate representation of the tooth structure.

For each tooth, a partial orthodontic fixed appliance was modeled, including a composite resin adhesive layer (0.2 mm thickness), which bonded the 0.022 × 0.028-inch rectangular MBT bracket and the 0.021 × 0.021-inch square Pitts 21™ self-ligating bracket system to the incisor's surface. Both types of brackets were designed based on CAD/CAM data, and archwires were passively inserted into the slots of the Pitts 21™ self-ligating bracket system and ligated using two steel ligatures in conventional brackets. The vertical positions of the brackets were standardized by placing them in the center of the middle third of the labial surface of the crown.

Finite element meshing

After constructing the 3D solid models, a finite element (FE) mesh was generated to ensure a node-to-node connection between the bracket, adhesive layer, tooth, PDL, and alveolar bone. A coarsening factor of 1.5 was used to ensure reliable meshing, while areas with complex geometry, such as the wire-slot interface, were finely meshed. The wire and ligatures were meshed separately from the bracket, allowing contact analysis based on Coulomb friction principles. A frictional coefficient of 0.2 was applied between the archwire and bracket to simulate realistic mechanical behavior. Second-order tetrahedral elements were used to discretize the bone, crown, root, PDL, bracket, archwire, adhesive, and ligature.

A total of 191,574 nodes and 119,965 elements were used to discretize model 1. For model 2, 192,765 nodes and 122,728 elements were utilized. This high-resolution meshing ensured an accurate representation of the mechanical interactions occurring at the wire-slot interface.

Material properties

The material properties of each component in the model were selected based on previous studies. All materials, including the tooth, bone, bracket, archwire, and adhesive, were modeled as homogenous and isotropic, except for PDL, which was treated as a bilinear elastic material. The material properties are listed in Table 1.

**Table 1:** Material properties used in the study PDL: periodontal ligament, MPa: megapascals, RMGI: resin-modified glass ionomer

Material	Young's modulus (MPa)	Poisson's ratio
Bone	2,000	0.3
PDL	Bilinear: 0.05/0.20 ultimate strain, ε12 7%	0.3
Tooth	20,000	0.3
Adhesive (composite resin)	8,823	0.25
Adhesive (RMGI)	7,600	0.30
Bracket (stainless steel)	200,000	0.30
Wire (stainless steel)	200,000	0.30

Simulation setup and boundary conditions

To simulate a clinical scenario of active palatal root torque, an incremental torque was applied to the archwires. Initially, the archwires were passively inserted into the base of the bracket slot before torque was applied. Boundary conditions were defined to restrict the movement of the apical bone surface while maintaining tight ligatures using a spring nodal tie. The torque applied to both ends of the archwire generates mechanical forces, inducing the labial movement of the root tip and the palatal movement of the crown. The resulting displacements of the crown and root, with the overall displacements, were calculated at the end of each simulation.

Sensitivity analysis

Sensitivity analysis was conducted to evaluate the reliability of the finite element mesh. By subdividing the elements across all three dimensions of a randomly selected model, the total number of elements was calculated. This additional analysis ensured that the mesh was sufficiently refined to capture all the necessary details of the bracket-archwire interaction. All simulations were conducted using HyperMesh 2019.0 software (Altair Engineering Inc., Troy, MI).

## Results

The highest torque expression, 13.8 N-mm, was observed in model 2 on the central and lateral incisors. For comparison, a torque expression of 10.68 N-mm was produced in model 1 on the same teeth. Crown displacement was also highest when the 0.020 × 0.020-inch SS wire was used in the 0.021 × 0.021-inch square pitch bracket on the central and lateral incisors. The torque expression increased in both models from 0° to 20°. There was a torque loss of 6.2° at 20° torque in the archwire, whereas it was 9.32° torque loss in model 1 (Table 2).

**Table 2:** Simulation results of torque expression in N-mm for the constructed models at various torque increments Model 1: conventional MBT rectangular bracket with a wire size of 0.019 × 0.025 inch, model 2: Pitts 21™ self-ligating bracket system with a wire size of 0.020 × 0.020 inch MBT: McLaughlin, Bennet, and Trevisi, N-mm: Newton-millimeter

Applied twist degree	Torque expression in N-mm	
	Model 1	Model 2
0°	0.00	0.00
5°	9.42	9.86
10°	10.12	11.68
15°	10.46	12.86
20°	10.68	13.80

A more precise fit was achieved with the 0.020 × 0.020-inch SS archwire in the 0.021 × 0.021-inch square self-ligating bracket compared to the 0.019 × 0.025-inch SS archwire in the 0.022 × 0.028-inch conventional MBT bracket. A crown displacement of 6.8° occurred with the 0.019 × 0.025-inch SS wire in the 0.022 × 0.028-inch conventional MBT bracket, while the 0.020 × 0.020-inch SS wire provided a more controlled displacement of 8.5° in the self-ligating square bracket at 20° torque. At all torque combinations, more controlled displacements were observed for the self-ligating brackets. Torque expression was better for the lateral incisors than for the central incisors (Table 3).

**Table 3:** Tipping angle in degrees for both models at different torque values Model 1: conventional MBT rectangular bracket with a wire size of 0.019 × 0.025 inch, model 2: Pitts 21™ self-ligating bracket system with a wire size of 0.020 × 0.020 inch MBT: McLaughlin, Bennet, and Trevisi

Torque in degrees (°)	Model 1	Model 2
0°	0°	0°
5°	1.8°	2.1°
10°	3.5°	4.2°
15°	5°	6.3°
20°	6.8°	8.5°

**Figure 1:**
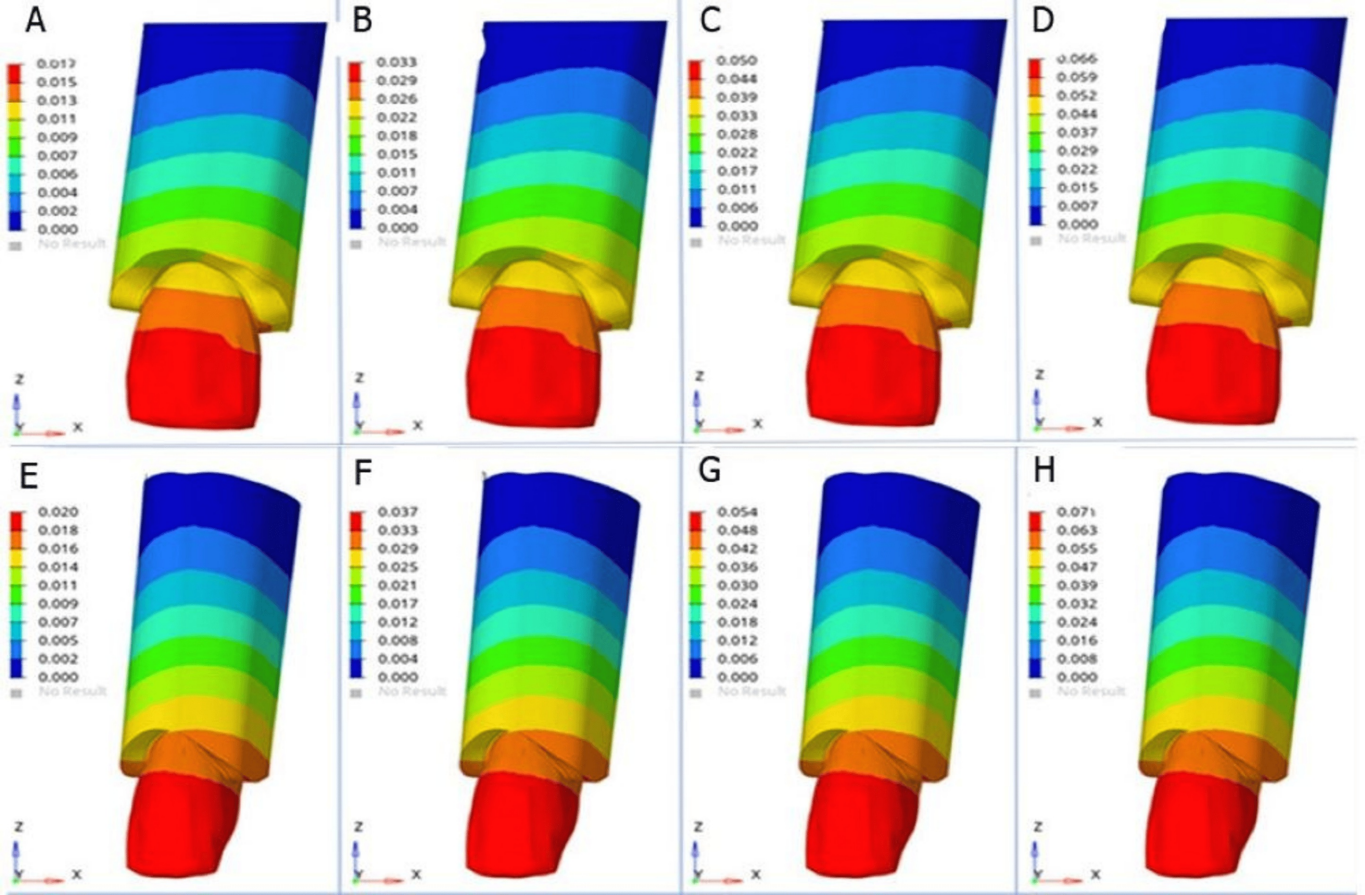
von Mises stress on maxillary central incisor and surrounding cortical bone at incremental torque (A) 5° torque in model 1, (B) 10° torque in model 1, (C) 15° torque in model 1, (D) 20° torque in model 1, (E) 5° torque in model 2, (F) 10° torque in model 2, (G) 15° torque in model 2, and (H) 20° torque in model 2 Model 1: conventional MBT rectangular bracket with a wire size of 0.019 × 0.025 inch, model 2: Pitts 21™ self-ligating bracket system with a wire size of 0.020 × 0.020 inch MBT: McLaughlin, Bennet, and Trevisi

**Figure 2:**
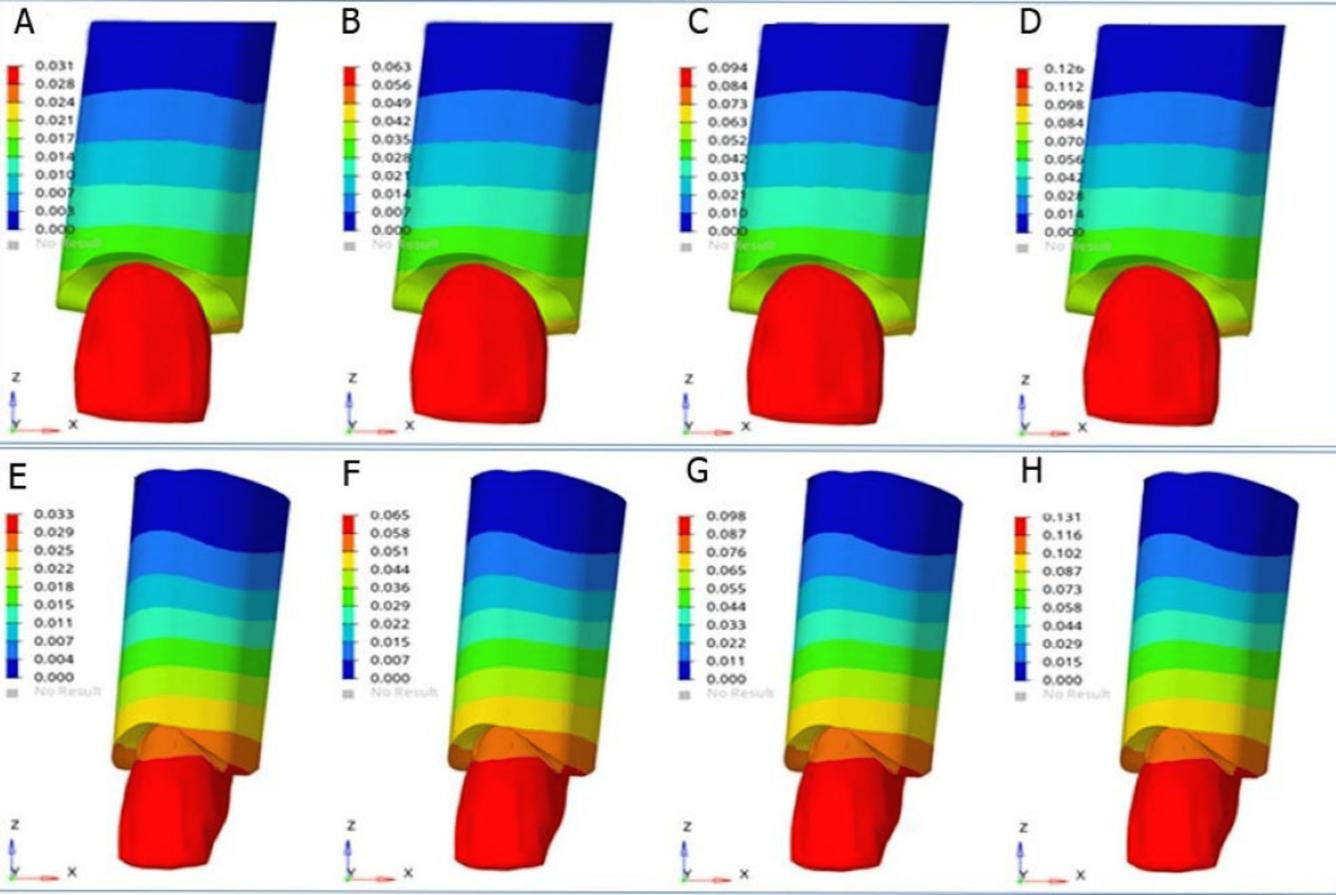
von Mises stress on maxillary lateral incisor and surrounding cortical bone at incremental torque (A) 5° torque in model 1, (B) 10° torque in model 1, (C) 15° torque in model 1, (D) 20° torque in model 1, (E) 5° torque in model 2, (F) 10° torque in model 2, (G) 15° torque in model 2, and (H) 20° torque in model 2 Model 1: conventional MBT rectangular bracket with a wire size of 0.019 × 0.025 inch, model 2: Pitts 21™ self-ligating bracket system with a wire size of 0.020 × 0.020 inch MBT: McLaughlin, Bennet, and Trevisi

The results of von Mises stress revealed that the highest von Mises stress on the maxillary incisors and cortical bone was observed at 20° torque in both models (Figure 1). Stresses were greater in the lateral incisors than in the central incisors (Figure 2).

Furthermore, it was revealed that the stresses were higher in model 2 for both the teeth and cortical bone than in model 1 (Table 4).

**Table 4:** von Mises stress on maxillary incisors and cortical bone in MPa MPa: megapascals

Torque in degrees (°)	Central incisor				Lateral incisor			
	Model 1		Model 2		Model 1		Model 2	
	Tooth	Cortical bone	Tooth	Cortical bone	Tooth	Cortical bone	Tooth	Cortical bone
5^o^	0.015	0.002	0.018	0.002	0.028	0.003	0.029	0.004
10^o^	0.029	0.004	0.033	0.004	0.056	0.007	0.058	0.007
15^o^	0.044	0.006	0.048	0.006	0.084	0.010	0.087	0.011
20^o^	0.059	0.008	0.063	0.008	0.112	0.014	0.116	0.015

**Figure 3:**
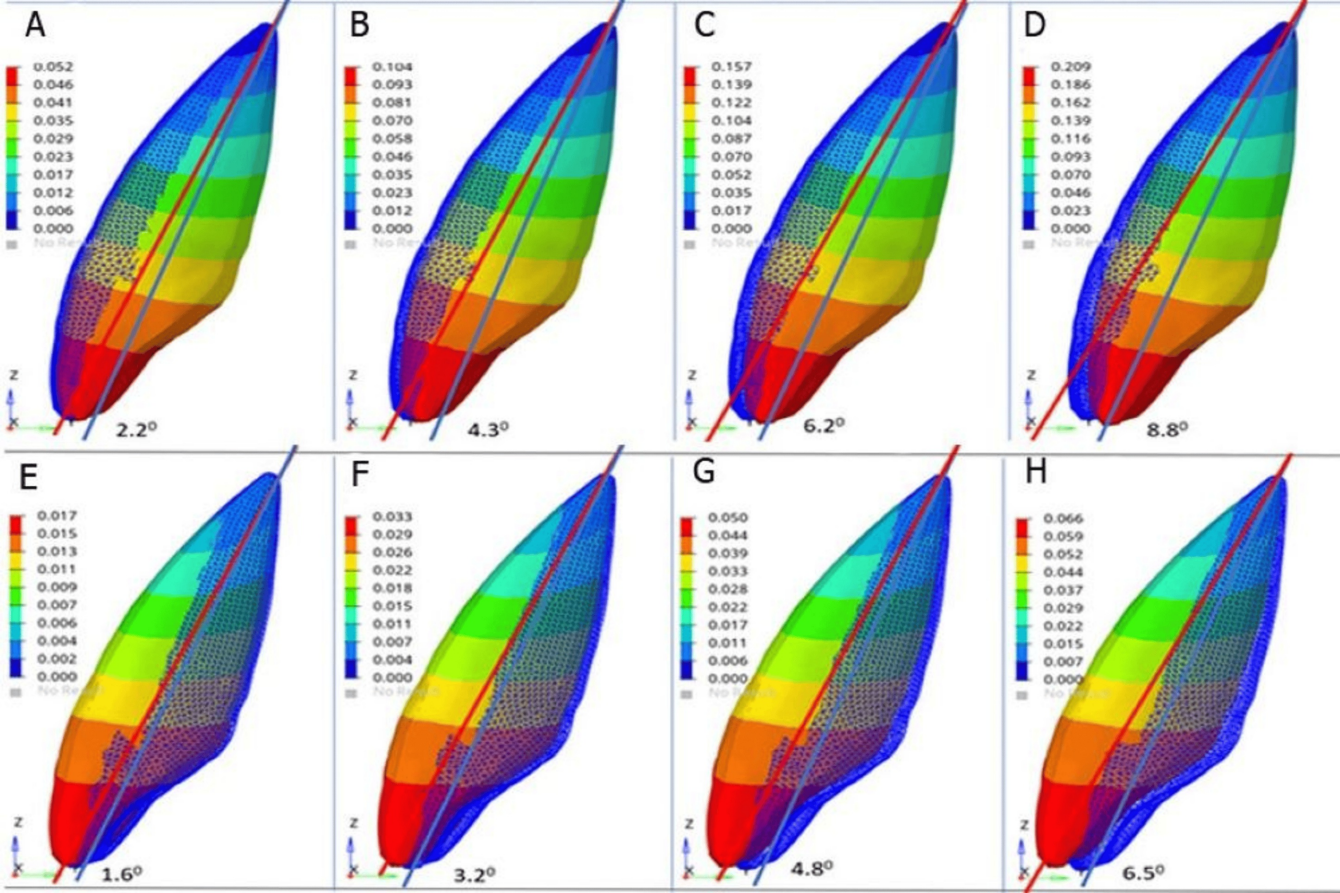
Displacement of maxillary central incisor at incremental torque (A) 5° torque in model 1, (B) 10° torque in model 1, (C) 15° torque in model 1, (D) 20° torque in model 1, (E) 5° torque in model 2, (F) 10° torque in model 2, (G) 15° torque in model 2, and (H) 20° torque in model 2 Model 1: conventional MBT rectangular bracket with a wire size of 0.019 × 0.025 inch, model 2: Pitts 21™ self-ligating bracket system with a wire size of 0.020 × 0.020 inch MBT: McLaughlin, Bennet, and Trevisi

More tipping movements were found in the central incisor than in the lateral incisor in both models. The tipping movements were greater in model 1 for all torque values than in model 2. The tipping movements were associated with labial tipping of the incisors, with slight palatal movement of the roots due to the palatal root torque placed in the archwire (Figure 3).

**Figure 4:**
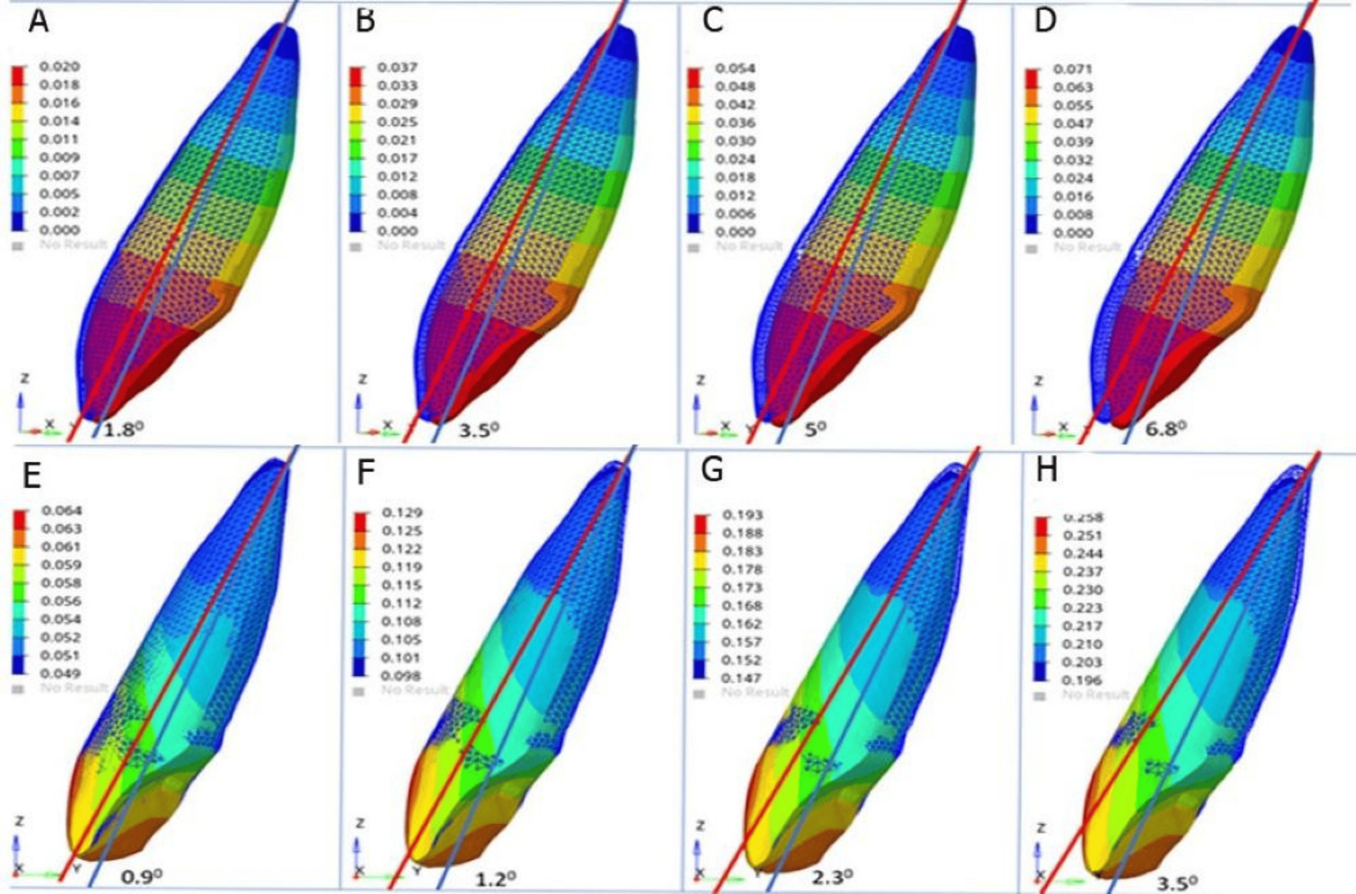
Displacement of maxillary lateral incisor at incremental torque (A) 5° torque in model 1, (B) 10° torque in model 1, (C) 15° torque in model 1, (D) 20° torque in model 1, (E) 5° torque in model 2, (F) 10° torque in model 2, (G) 15° torque in model 2, and (H) 20° torque in model 2 Model 1: conventional MBT rectangular bracket with a wire size of 0.019 × 0.025 inch, model 2: Pitts 21™ self-ligating bracket system with a wire size of 0.020 × 0.020 inch MBT: McLaughlin, Bennet, and Trevisi

The highest labial tipping was observed at 20° torques and the lowest at 5°. This showed that increased incremental torque was associated with increased proclination of the lateral incisors (Figure 4), and this movement was more pronounced for the central incisor and model 1 (Table 5).

**Table 5:** Displacement in degree in both the models Model 1: conventional MBT rectangular bracket with a wire size of 0.019 × 0.025 inch, model 2: Pitts 21™ self-ligating bracket system with a wire size of 0.020 × 0.020 inch MBT: McLaughlin, Bennet, and Trevisi

Torque in degrees (°)	Displacement of central Incisor in degrees (°)		Displacement of lateral Incisor in degrees (°)	
	Model 1	Model 2	Model 1	Model 2
5^o^	2.2^o^	1.6^o^	1.8^o^	0.9^o^
10^o^	4.3^o^	3.2^o^	3.5^o^	1.2^o^
15^o^	6.2^o^	4.8^o^	5^o^	2.3^o^
20^o^	8.8^o^	6.5^o^	6.8^o^	3.5^o^

## Discussion

While a multitude of prior research endeavors concerning torque moments and their expressions have predominantly concentrated on the evaluation of pre-treatment and post-treatment values for dental structures, as represented in digital models, utilizing a defined tooth coordinate system for analysis, our investigation has adopted a significant more extensive approach through the implementation of FEA. This advanced methodology has facilitated a thorough examination of the torque characteristics exhibited by orthodontic brackets within a meticulously controlled environment, thereby enabling us to deliver a more accurate and nuanced assessment of their biomechanical performance through the use of sophisticated simulation techniques. Furthermore, the application of finite element modeling presents an invaluable opportunity to predict the behavioral responses of biological structures that are integral to the specific conditions outlined in our study, thereby enriching the overall understanding of these complex interactions. It is also noteworthy that measurements that cannot be feasibly obtained in vivo owing to various practical constraints may still yield critical insights that are beneficial for clinical investigations, thus establishing the relevance and importance of FEA in our research framework.

The results of the present study suggested that model 2, using the 0.020 × 0.020-inch SS wire with a 0.021 × 0.021-inch Pitts bracket, demonstrated higher torque expression (13.8 N-mm) on maxillary incisors than model 1 with conventional MBT brackets (10.68 N-mm). The increased crown displacement and torque expression in model 2 indicate superior control and engagement between the wire and bracket, leading to more efficient tooth movement. Model 2 exhibited a smaller play of 6.2° than model 1 (9.32°) at 20° of activation, further reinforcing the mechanical advantage of model 2. Our findings were in agreement with a study by Satapathy et al., who concluded that square brackets with square archwire produced better torque expression than rectangular brackets with rectangular wires [6]. The square slot configuration signifies a significant shift from the conventional rectangular slot designs generally employed in orthodontic brackets. This square slot configuration presents several benefits, such as facilitating a more accurate and regulated system for adjustments related to torque and tooth inclination throughout the orthodontic process. The initial control of the tip and torque is enhanced by utilizing archwires that possess a square cross-section within a square slot, thereby allowing for rapid complete slot engagement during treatment. The implementation of variable modulus technology permits the earlier use of softer, full-sized wires in treatment, thus providing enhanced 3D control [7]. The square slot secures the archwire in place, even amidst rotational motion and retraction of the anterior dentition [8].

Our study further revealed that stress was greater in lateral incisors than in central incisors. This finding is supported by those of previous studies [3,9]. This could be due to the smaller root surface of lateral incisors exhibiting increased stresses at the same torque values as central incisors with a greater root surface area. Our study further indicated that stresses were greater with square archwire in square passive self-ligating brackets, compared to rectangular archwire in rectangular brackets. This finding is in agreement with a previous study [6]. This could be due to the fact that passive self-ligating brackets are engineered to minimize friction; however, when utilized with square archwires, the heightened snugness or interaction with the bracket walls may result in phenomena known as "binding" in specific regions, consequently leading to the concentration of stress [10]. In contrast, rectangular archwires provide enhanced mobility and exhibit diminished engagement with bracket walls [11]. The geometric configuration of square archwires typically renders them stiffer in terms of their rotation. This inherent stiffness can lead to resistance of the archwire to rotational movements or adjustments, thereby imposing increased stress on the bracket, particularly during torque control or angular corrections [9].

Our study also indicated that more controlled tooth movements were associated with model 2 than with model 1. This could be due to a more precise fit of the 0.020 × 0.020-inch SS archwire in the 0.021 × 0.021-inch square self-ligating bracket, compared to the 0.019 × 0.025-inch SS archwire in the 0.022 × 0.028-inch conventional MBT bracket. Less play associated with precise slot engagement led to controlled torquing movements with less unwanted tooth movement, such as proclination of the incisors. It has been recommended in a systematic review by Archambault et al. that for effective torque expression, stiffer, higher-dimension SS wires should be used such as 0.021 x 0.021-inch SS wire in a 0.022 x 0.028-inch slot [2]. However, we used a 0.019 x 0.025-inch wire in a 0.022 x 0.028-inch slot, which is the most commonly used wire in rectangular slots, which might have led to the increased play observed in our study. According to a study by Papageorgiou et al., it was concluded that rectangular archwires produce less torquing moments in 0.022 x 0.028-inch slots [12].

The higher torque moments observed in our study in model 2 align with the results of a previous study, where it was concluded that the elevated torque moment values linked to the Pitts 21™ brackets could provide enhanced torque regulation, rendering them especially appropriate for orthodontic procedures that necessitate the alignment of teeth exhibiting significant angular deviations [6]. However, our results contradict those of a study by Dalstra et al. [13] and Morina et al. [14] who found that conventional brackets with steel ligatures pressed the archwire close to the slot, leading to increased torque expression compared to passive self-ligating brackets. This variation could have been due to the difference in the archwire-slot combination used in their study, as they used 0.019 x 0.025-inch SS wire in a 0.022 x 0.028-inch slot for both conventional and self-ligating systems. A systematic review by Al-Thomali et al. also concluded that conventional brackets show better torque expression than self-ligating brackets [15]. However, the studies included in their systematic review used a variety of archwire-slot combinations, leading to variability in the results.

Clinical implications of our study

The unique contribution of our study to this field of knowledge is underscored by the fact that it represents the inaugural effort to systematically evaluate and compare torque expression between square and rectangular slot brackets, specifically within the context of a labial orthodontic appliance. For better torque expression with reduced play, a square wire in square Pitts 21™ passive self-ligating brackets is preferable to rectangular wires in conventional brackets. However, to reduce stress on the tooth and cortical bone, a rectangular wire can also be used in square Pitts 21™ brackets.

Limitations

The constraints inherent in this FEA investigation include the reduction of intricate biological architectures, including the anisotropic characteristics of bone, and the omission of soft tissues, such as the PDL, which is critical in the force dissipation mechanisms observed in natural dentition. The model further presupposes idealized and homogenous material properties along with consistent loading conditions, which may inadequately represent the heterogeneity present in clinical environments. Moreover, individualized factors pertaining to patients, such as bone quality, implant positioning, and unique occlusal forces, were not integrated into the analysis. These simplifications restrict the applicability of the findings to real-world scenarios specific to individual patients [16,17].

## Conclusions

Based on the results of the present study, it was concluded that the Pitts 21™ passive self-ligating system produced better torque expression and less play with square SS archwire compared to conventional brackets with rectangular SS archwire. Better torque expression was observed at higher torque values. However, the corresponding stresses on the teeth and cortical bones also increased. Higher stresses were observed with Pitts 21™ brackets than with conventional brackets. Nonetheless, it is crucial to acknowledge that the findings derived from FEA analyses do not accurately replicate in vivo conditions. It is advisable that the outcomes of this investigation be validated through clinical trials to authenticate the results. Nevertheless, these findings enhance our understanding of the mechanical properties of brackets and archwires, providing clinicians with valuable insights to attain optimal results.
